# Correctors modify the bicarbonate permeability of F508del-CFTR

**DOI:** 10.1038/s41598-020-65287-4

**Published:** 2020-05-21

**Authors:** Michele Fiore, Cristiana Picco, Oscar Moran

**Affiliations:** Istituto di Biofisica, Consiglio Nazionale delle Ricerche. Via De Marini, 6, 16149 Genova, Italy

**Keywords:** Biological techniques, Biophysics, Cell biology, Drug discovery

## Abstract

One of the most common mutations in Cystic Fibrosis (CF) patients is the deletion of the amino acid phenylalanine at position 508. This mutation causes both the protein trafficking defect and an early degradation. Over time, small molecules, called correctors, capable of increasing the amount of mutated channel in the plasma membrane and causing an increase in its transport activity have been developed. This study shows that incubating *in vitro* cells permanently transfected with the mutated channel with the correctors VX809, VX661 and Corr4a, and the combination of VX809 and Corr4a, a recovery of anion transport activity is observed. Interestingly, the permeability of bicarbonate increases in the cells containing corrected p.F508del CFTR channels is greater than the increase of the halide permeability. These different increases of the permeability of bicarbonate and halides are consistent with the concept that the structural conformation of the pore of the corrector-rescued p.F508del channels would be different than the normal wild type CFTR protein.

## Introduction

Cystic fibrosis (CF), caused by mutation in Cystic Fibrosis Transmembrane Regulator (*CFTR*) gene^1,2^, is one of the most common autosomal recessive lethal inherited diseases in the Caucasian poulation^3,4^. The CFTR protein is an ATP-binding cassette transporter with two membrane-spanning domains (MSD1 and MSD2), two nucleotide binding domains (NBD1 and NBD2) and a unique regulatory domain (R)^5^. The protein works as an ATP- and phosphorylation-regulated anion channel^6^, that is permeable to the halides (Br^−^, Cl^−^, I^−^, F^−^)^7,8^, and can also conduct HCO^-^_3_ with a ratio of the permeability between bicarbonate and chloride (*P*_*HCO3*_*/P*_*Cl*_*)* of ~0.25^9–11^. The physiological role of CFTR is the maintenance of fluid transport across epithelial cells of airways, intestines, pancreatic as well as bile ducts^12^. The CF respiratory disease is the main cause of death and the main determinant of the burden on the quality of life. Most of the health problems in CF respiratory disease can be attributed to the viscous mucus phenotype, hindering ciliary activity and their clearance mechanism^13^. This pathogenicity is caused by the malfunctioning of the CFTR channel which does not adequately transport chloride and bicarbonate across the plasma membrane causing lack of ions and water homeostasis at the surface of the airway epithelia^14,15^.

To date, more than 2,000 mutations have been identified that cause CF, classified in six classes according to the effect of the mutation leading to anion transport defects in epithelia^16^. The most common mutation is the deletion of phenylalanine 508, which is located in the NBD1 (p.F508del, class II mutation), causing aberrant assembly of the full-length protein and making the channel susceptible to premature degradation via the protein quality control mechanism^16,17^. The p.F508del-CFTR protein is a functional anion channel, but it is unstable and rapidly degrading, leading to a great reduction of chloride and bicarbonate transport by the CFTR channel^18–20^.

In the CF patients, the reduction of bicarbonate transport gives rise to a lower pH in airways surface liquid (ASL)^15,21^ and interferes with the ASL fluidity. The ASL is composed of mucus and periciliary liquid layer (PLC) and is very important to keep intact the defense mechanisms of the respiratory airway. Low concentration of HCO^-^_3_ may interfere with the post-secretory modifications of the mucins MUC5AC and MUC5B^15,22^. When the mucus retains the physiological characteristics, the cilia of the airways epithelium are able to discharge the mucus containing potential pathogens captured during breathing^22^.

As a feasible therapy for CF patients carrying the mutation p.F508del, small molecules, named correctors, that are able to increase the amount of protein in the plasma membrane have been developed^23,24^. It has been proposed that the molecules capable of correcting the mutated protein can be divided into three classes. Class 1 is represented by molecules that are likely to interact with the NBD1; class 2 is represented by molecules that would interact with the NBD2; class 3 correctors would facilitate the folding of the NBD1 segment or prevents it from being denatured^25–27^.

Here we examine the halide and bicarbonate permeability of the p.F508del-CFTR rescued by three correctors, VX809 and VX661, that belong to the class 1, and Corr4a, of class 2. We show that the rescued p.F508del-CFTR has a higher bicarbonate relative permeability than the wild type control. These results have important implications in the correctors-based CF therapeutic strategy.

## Methods

### Cell cultures

Fisher Rat Thyroid (FRT) cells stably transfected with a wild type (WT) CFTR, or CFTR carrying the cystic fibrosis mutation p.F508del, were grown at 37 °C and 5% CO_2_ in modified F12 Coon’s medium with addition of 10% FBS and, 2 mM of Glutamine, 1 mg ml^−1^ penicillin, 100 µg ml^−1^ streptomycin and the addition of 1 mg ml^−1^ geneticin (G418) and 0.6 mg ml^−1^ zeocin as selection agents. For the iodide influx assay, cells stably co-transfected with the halide-sensitive yellow fluorescent protein, YFP-p.H148Q/p.I152L^28,29^, were seeded in 96-well microplates at a density of 40,000 cells/well. For intracellular pH measurements, cells were seeded on glass-bottom Petri dishes. Measurements were carried out 48 h after seeding. To evaluate the correctors p.F508del rescue on the cells expressing the mutated CFTR, cells were incubated for 18 h with 5 µM of VX809 (lumacaftor; Selleck Chemicals, Huston, TX, USA), VX661 (tezacaftor; Selleck Chemicals), Corr4a (ChemBridge Corp. San Diego, CA, USA), or 2.5 µM of VX809 with 2.5 µM of Corr4a together (final concentration 5 µM). Except when indicated, all chemicals compounds were purchased from Sigma-Aldrich (Milan, Italy).

### Iodide Influx assay

To evaluate the halide transport activity of the CFTR channels, cells were incubated for 30 minutes with 20 µM forskolin at 37 °C in a solution containing (in mM): KNO_3_ 4.5, Ca(NO_3_)_2_ 1.2, MgSO_4_ 0.2, Glucose 5, HEPES 20, pH 7.4 NaCl 136, in a final volume of 60 µL. Thus, we measured the influx of iodide, which causes the quenching of the YFP as enters into the cell. The fluorescence of YFP was monitored using a fluorescence plate reader (Tristar2 S, Berthold Technologies, Bad Wildbad, Germany), equipped with 485 nm excitation and 535 nm emission filters^30,31^.

After recording the fluorescence for 5 seconds as a baseline, cells are perfused with 100 µL of a solution where NaCl was substituted by NaI, and fluorescence was monitored every 0.2 seconds for a further 30 seconds. In this way, the final concentration of NaI in the bath was 85 mM. Measurements were performed at 37 °C.

The fluorescence time course was normalized by the average of the fluorescence of the baseline recorded before NaI injection. The initial rate of fluorescence decay (QR) was derived by fitting the signal with a double exponential function. The QR is an indicator of halide transport by the CFTR^28,31^.

### Bicarbonate transport in cells

The transport of bicarbonate was evaluated by measuring the variation of the intracellular pH (pH_i_) following the NH_4_ ^+^ prepulse technique^31,32^. The pH_i_ was measured in FRT cells using the fluorescent pH indicator 2′,7′-bis-2-(carboxyethyl)-5-(and6) carboxyfluorescein ester (BCECF-AM; Thermo Fisher Scientific, Waltham, MA USA). Cells were loaded with 5 μM of BCECF-AM in the culture medium without serum for 30–40 min at room temperature. After loading, cells were washed two times with the recording solution containing (mM): NaCl 140, K_2_HPO_4_ 2.5, MgSO_4_ 1, CaCl_2_ 1, HEPES 10, glucose 6 (pH 7.3) and were allowed to recover for at least 30 min before measurement. The Petri dish was mounted in a perfusion system in the stage of an epifluorescence inverted microscope (iMIC) with a Qimaging Retiga EXI Blue camera (Till Photonics, Graefelfing, Germany). During the experiments, the perfusion solutions were equilibrated with 5% CO2 and 95% air.

Cells were visualized with an Objective Plan Super Apochromat 10x (Olympus, Tokyo, Japan; N.A. 0.4, W. D. 30.1 mm). For excitation, we used Till Oligochrome (FEI, Munich, Germany), a wavelength switching device containing a stable xenon light source. The sample was excited at two wavelengths, 440 nm and 490 nm, and emission was recorded at 520 nm. To calibrate the pH_i_ measurements, the pH_i_ was varied incubating the cells in a high potassium concentration solution with 15 μM nigericin to equilibrate the intracellular compartment with various external pH values; a calibration curve was constructed plotting the pH against the ratio of fluorescence emitted upon excitation at the two excitation wavelengths^31^.

The NH_4_^+^ solution was prepared by replacing 30 mM NaCl in the standard recording solution with an equimolar concentration of NH_4_Cl. When cells were subjected to an acid load by the transient application (2–3 min pulse) of a 30 mM NH_4_^+^ solution, the pH_i_ rose as NH_4_^+^ accumulated in the intracellular space during the NH_4_Cl perfusion. Cells were subsequently returned to a recording solution without NH_4_^+^, and acidification of the cytoplasm occurred when NH_3_ quickly diffused out of the cell. For the bicarbonate recording solution, 30 mM of NaCl was substituted by NaHCO_3_. The Na^+^/H^+^ exchange and the Na^+^/HCO_3_^−^ co-transport were inhibited with 1 mM amiloride, and the Cl^−^/HCO_3_^−^ and the Na^+^-dependent Cl^−^/HCO_3_^−^ exchangers inhibited by adding 300 μM disodium 4,4′-diisothiocyano-2,2′-stilbenedisulfonate (DIDS) to the recording solution^33–35^. Moreover, DIDS also inhibits the calcium-activated chloride channels bestrophin and anoctamin-1 that both display HCO_3_^−^ permeability^36^. CFTR was activated by the addition of 100 µM of the permeable cAMP analog pCPT-cAMP to the recording solutions. The HCO_3_^−^ influx was calculated from the pH_i_, applying the Henderson-Hasselbach equation and assuming a constant CO_2_ partial pressure. Details of the HCO_3_^−^ flux calculations are presented in the supplementary file.

### Data analysis

The time course of the recorded fluorescence traces, as well as the statistical analysis, was evaluated using IgorPro (version 8.03, Wavemetrics, Portland, Oregon). Results are presented as mean ± standard error (SEM) and the number of measurements. Statistical analysis was performed using Student’s t-test or one-way ANOVA to compare the different data sets; *P* values < 0.05 were considered statistically significant.

## Results

**Figure 1:**
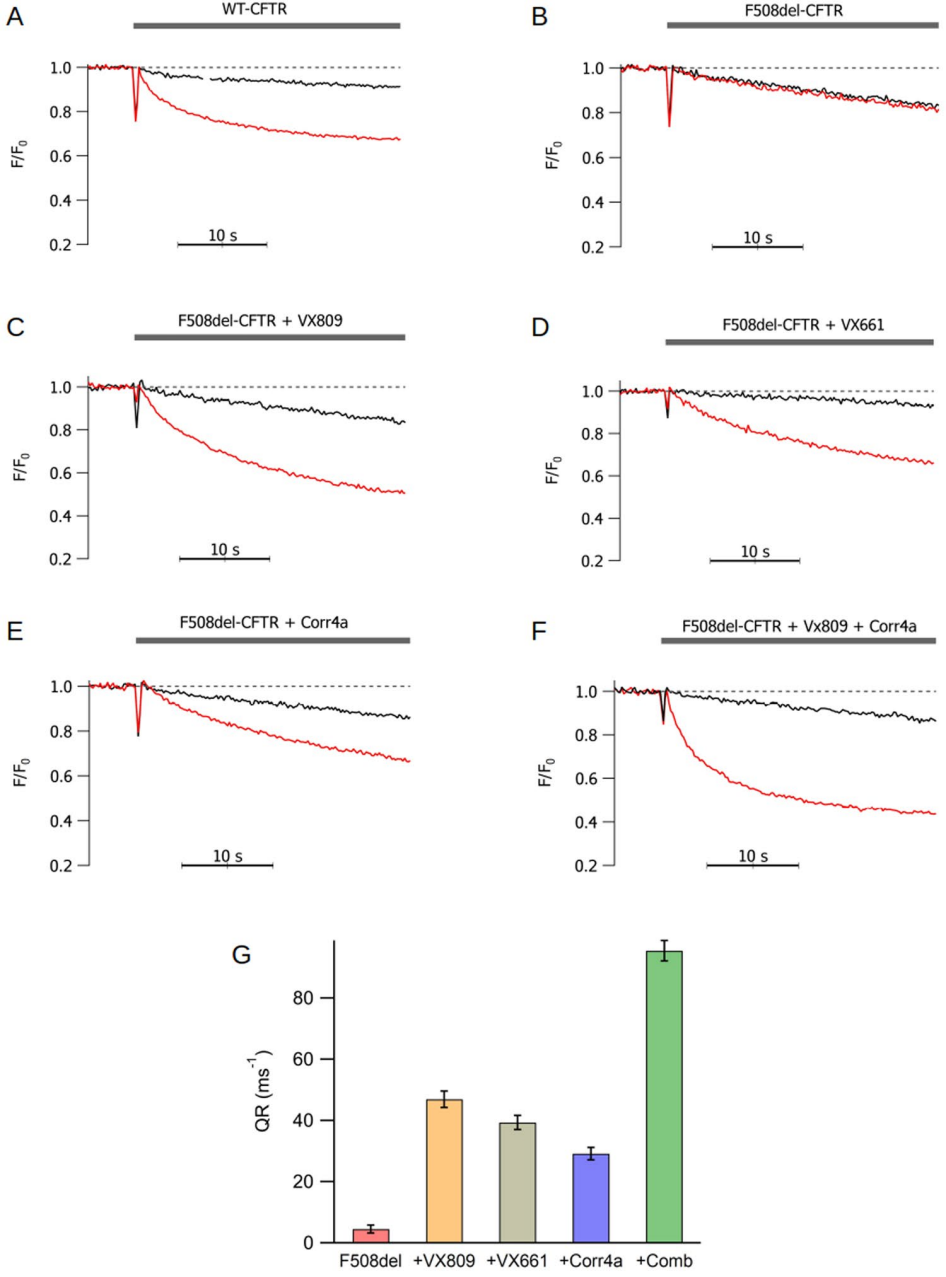
Iodide influx assay in FRT cells. Time course of the iodide-sensitive YFP fluorescence decay, normalized by the initial fluorescence, after the addition of 85 mM of NaI in the external solution of WT-CFTR cells (**A**) and p.F508del-CFTR cells (**B–F**) before (black traces) and upon the addition of 20 µM of forskolin to activate the channels (red trace). Panels (**B**–**F**) represent respectively the cells not incubated, incubated with VX809, VX661, Corr4a and the combination of VX809 and Corr4a together. The number of measures for each condition was between 9 and 12. The **G** panel is the representation of the summary of the experiment that shows the change produced by each corrector. Data are the average +/− SEM. The number of the experiment in each condition was between 9 and 12.

The iodide influx was measured as the initial quenching rate of the fluorescence, QR, in FRT cells expressing halide-sensitive YFP and WT-CFTR or the mutant p.F508del-CFTR, respectively. Application of 20 µM of forskolin elicited the fluorescence decay after the addition of iodide to the external solution (QR = 62.9 ± 1.4 ms^−1^; n = 10), implying iodide influx driven by the activation of the WT-CFTR channels (Fig. 1A). Similar experiments were carried out on cells expressing p.F508del-CFTR, where the application of 20 µM of forskolin elicited a very small iodide transport (QR = 4.6 ± 0.8 ms^−1^; n = 12). Incubation of the p.F508del-CFTR cells with 5 µM of VX809, VX661, or Corr4a results a significant increase of the iodide influx elicited by forskolin, yielding a QR of 47.0 ± 2.6 ms^−1^ (n = 12), 39.4 ± 2.3 ms^−1^ (n = 9) and 29.2 ± 1.4 ms^−1^ (n = 12), for VX809, VX661 and Corr4a, respectively (Fig. 1B–E). The treatment of the cells with a combination of 2.5 µM VX809 and 2.5 µM Corr4a results in a further increase of the iodide transport, yielding a QR of 95.5 ± 2.9 ms^−1^ (n = 12), according to previous studies in which the combination of the two correctors has a synergistic activity (Fig. 1F)^25–27,37^. The graph in Fig. 1G shows a summary of all the QR values of the iodide influx experiments.

**Figure 2:**
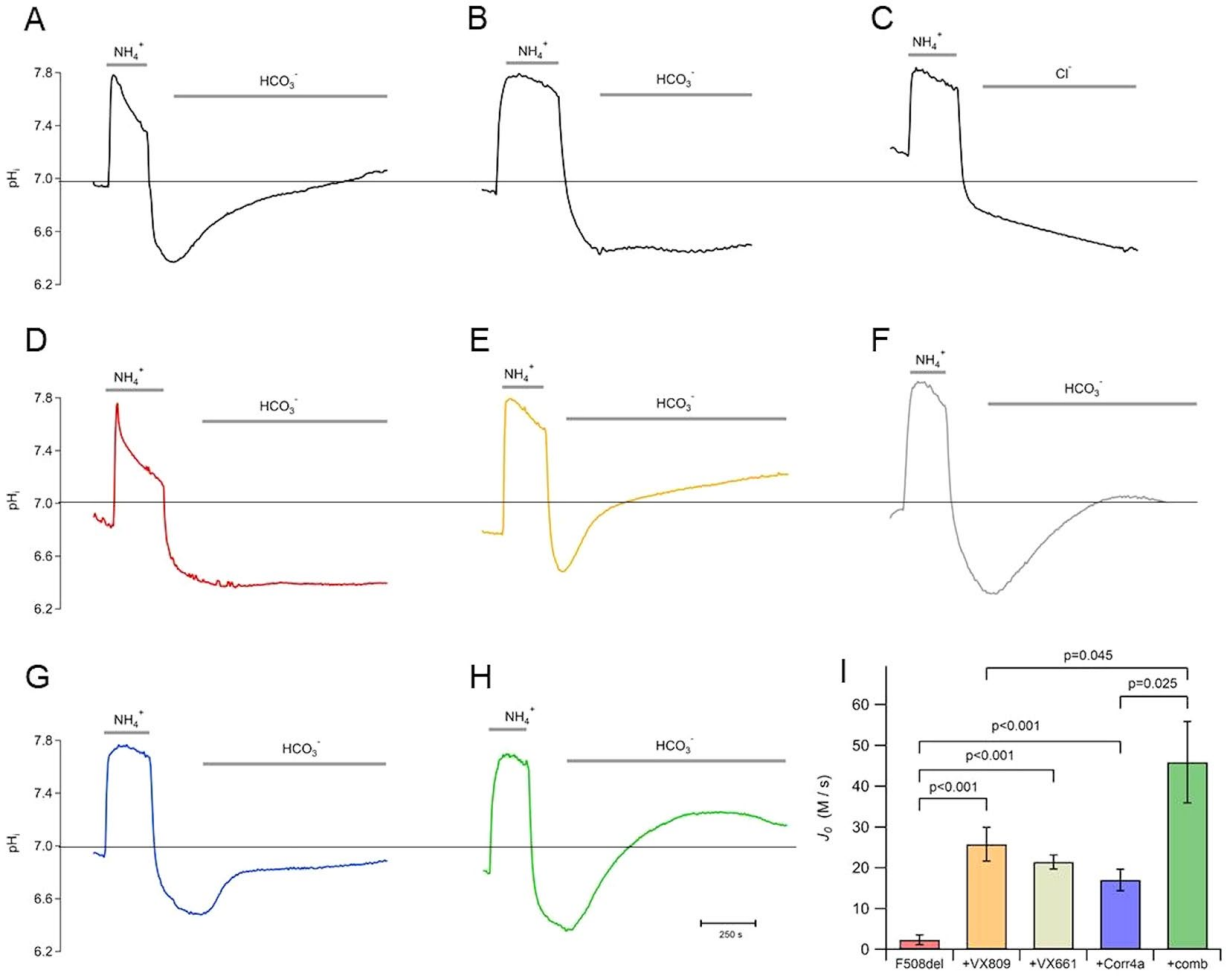
Intracellular pH measurement using ammonium pulse protocol (see methods). Perfusion of FRT WT-CFTR and F508del-CFTR cells with 30 mM of NH^+^_4_ causes the augment of the pH_I_ followed by rapid acidification when NH^+^_4_ is removed. When bicarbonate was perfused an alkalinization was observed upon the 100 µM pCPT-CAMP stimulation in FRT WT-CFTR (**A**); FRT WT-CFTR without pCPT-CAMP stimulation or with Cl^−^ in place of HCO_3_^−^ was used as control (**B**,**C**). FRT p.F508del-CFTR cells not incubated with correctors do not elicit any pH_i_ variation and consequent bicarbonate transport (**D**), while incubation with VX809, VX661, Corr4a and the combination of VX809 and Corr4a, gives rise to an HCO_3_^−^ influx (**E–H**). The panel **I** show the summary of bicarbonate influx of p.F508del-CFTR not incubated (red); incubated with VX809 (orange), VX661 (gray), Corr4a (blue) and the combination (green) respectively. Data are means ± SEM. The number of experiments for each condition was between 8 and 12.

The transport of bicarbonate through the CFTR channels was evaluated measuring the intracellular pH response to the NH_4_^+^ pulse protocol^31,32^. The washout of NH_4_^+^ from the solution induces acidification of the cells; the restore of neutral pH_i_ is observed when HCO_3_^−^ is added to the bath and WT-CFTR is activated by pCPT-cAMP (Fig. 2A). The addition of DIDS and amiloride to the perfusion solutions assures that other HCO_3_^−^ pathways are blocked, and therefore the intracellular alkalinization is due to the entrance of HCO_3_^−^ through the activated CFTR. Indeed, the alkalinization of the cells is not present when the CFTR was not activated by the cyclic nucleotide (Fig. 2B). Similarly, when the cells were perfused with an HCO_3_^– ^free solution, even if the CFTR was activated by pCPT-cAMP, no pH_i_ variation was observed (Fig. 2C). These results show that the intracellular alkalinization of the cells expressing WT-CFTR is due to the HCO^−^_3_ influx through the CFTR channels.

When the experiments were carried out in cells expressing p.F508del-CFTR, in presence of HCO_3_^−^, the activation of the mutant CFTR by pCPT-cAMP elicited a very small pH_i_ variation after the NH_4_^+^ pulse, that corresponds to an HCO_3_^−^ influx of 2.4 ± 1.2 µM/s (n = 10) (Fig. 2D). Conversely, the treatment with correctors rescues the p.F508del-CFTR activity, responding to the post-pulse HCO_3_^−^ perfusion with an intracellular alkalinization, yielding an HCO_3_^−^ influx of 25.8 ± 4.1 µM/s (n = 12), 21.5 ± 1.7 µM/s (n = 9) and 17.0 ± 2.6 µM/s (n = 10) for the cells treated with VX809, VX661 and Corr4a, respectively (Fig. 2E–G). Similarly, as the data obtained measuring the halide transport, when cells expressing p.F508del-CFTR are treated with a mixture of the two correctors (VX809 + Corr4a), a faster alkalinization is observed consistent with an augmented HCO_3_^−^ influx, 45.9 ± 10.0 µM/s (n = 8) (Fig. 2H). These data confirm the synergistic effect of VX809 and Corr4a observed also in the iodide influx experiments. A summary of the quantitative evaluation of the HCO_3_^−^ influx is shown in Fig. 2I.

Tables with the experimental data and the statistical comparisons are shown in the supplementary file.

## Discussion

The main purpose of this study was to observe the pharmacological effect of correctors, individually or combined, on the p.F508del-CFTR channel permeability. Hence, we evaluated the halide and the HCO_3_^−^ transport driven by the p.F508del-CFTR channels rescued by three different correctors, VX809, VX661 and Corr4a. To avoid possible interferences of potentiators with the correctors-rescue of CFTR^38,39^, we have not applied any potentiator during the functional assay. The halide transport was done by measuring the kinetics of the fluorescence quenching of iodide-sensitive YFP. This method has been proven to yield data that are proportional to the Cl^−^ transport capacity of the CFTR^29,40^. Thus, we observed that FRT cells permanently transfected with halide sensitive YFP and p.F508del-CFTR showed a very small iodide transport evoked by forskolin. Indeed as previously demonstrated, the p.F508del-CFTR mutation gives rise to a functional channel with a wrong conformation, that brings to rapid degradation, but only a small amount of channels reach the membrane^18–20^. The incubation of these cells with VX809, VX661 or Corr4a rescues of the mutated proteins and a greater expression of the channel on the membrane surface^22,41–43^, resulting in a significant increase of iodide transport by the rescued p.F508del-CFTR. Interestingly, while the incubation with VX809, VX661 or Corr4a yielded a 10.3, 8.6 and 6.4-fold increase of halide transport, respectively, the use of VX809 and Corr4a together produced a significantly larger 20.8 fold-increase, indicating a synergistic effect of the two correctors. These results are similar to those recently reported elsewhere^43^.

Since it is not possible to directly evaluate the transport of bicarbonate, we used the ammonium pulse technique^31,32^. We first demonstrated that, in our experimental conditions, the post-pulse alkalinization of the cytoplasm requires the activation of the CFTR and the presence of extracellular HCO_3_^−^. Hence, the HCO_3_^−^ influx in corrector-rescued p.F508del-CFTR cells was calculated from the post-pulse alkalinization rate. For the p.F508del-CFTR, the increase of the HCO_3_^−^ influx is similar to that observed for the halide transport, yielding 10.9, 9.1, 7.2 and 19.5-fold increase for VX809, VX661, Corr4a, and the combination of VX809 and Corr4a, respectively. The linear correlation test of the HCO_3_^−^ influx and halide transport data of the rescued p.F508del-CFTR yields a significant correlation (*r* = 0.89, *P* < 0.0004). It indicates a similar ratio between the HCO_3_^−^ influx and halide transport measured in the corrector-rescued p.F508del-CFTR of 0.54 ± 0.02.

The FRT cells co-expressing the iodide-sensitive YFP protein were obtained from the clones previously transfected with the WT- or F508del-CFTR genes. As a result, we can assume that the protein levels of CFTR genes between YFP and no YFP cell lines are identical. However, because the WT- and F508del-CFTR transfected cell lines are two different clones, the number of plasmid copies cannot be considered identical a priori. Consequently, it is not possible to compare the absolute values of the functional assays of ion transport between these CFTR isoforms with our preparation. However, independently from the absolute values, the HCO_3_^−^ influx and halide transport ratio will reflect the HCO_3_^−^/halide permeability ratios. Interestingly, the WT-CFTR has an HCO_3_^−^ influx and halide transport ratio of 0.23, which is significantly smaller than that of the corrector treated p.F508del-CFTR (*P* < 0.0008). The augmented HCO_3_^−^ influx and halide transport ratio of the corrector-rescued mutant-CFTR can be explained with a decrease of the halide permeability, or the increase of the HCO_3_^−^ permeability. We discard a reduction of the halide permeability by correctors treatment since different reports have indicated that the absolute halide permeability of rescued p.F508del channels is similar to that of the WT-CFTR^44–46^. Therefore, most probably there is an increase of the HCO_3_^−^ permeability in the rescued mutants. This result for the VX809 is similar to that previously reported^11^; for the other two correctors, VX661 and Corr4a, this is the first report regarding the permeability of the rescued mutant-CFTR.

When human bronchial epithelia from CF-patients with the mutation p.F508del are treated with VX809 there is a reduction of the mucus viscosity, but there is not a significant effect in the fluid re-absorption by the epithelia^47^. Since the homeostasis of the ionic content of the ASL is regulated by the CFTR-secretion of Cl^−^, the lack of fluid reabsorption can be explained by a reduced Cl^−^ secretion due to an incomplete mutant rescue. However, the increased HCO_3_^−^ permeability of the rescued p.F508del-CFTR would favor the HCO_3_^−^ secretion, facilitating the post-secretional modification of the mucin, and the consequent reduction of the viscosity.

In summary, we showed that correctors of class 1, VX809 and VX661, a corrector of class 2 Corr4a, or a combination of the two classes of correctors, increase both halide and HCO_3_^−^ transport on cells transfected with the p.F508del-CFTR channels. However, the rescued p.F508del-CFTR has a bigger relative bicarbonate permeability, independently to the corrector class. Thus, we expect that a combination of two class 1 correctors, nowadays used for CF treatment^50^, will not modify this higher bicarbonate permeability pattern. A difference in the ions permeability would reflect a different structure of the ion pore in the channel. Structural studies have revealed differences between WT and mutated proteins^48,49^. Paradoxically, this incomplete rescue of the p.F508del-CFTR may represent a therapeutical advantage, as the enhanced HCO_3_^−^ secretion would favor the mucus fluidification.

The encouraging results obtained in this work show the need to use cell lines that endogenously express CFTR, both wild type and mutated, such as a bronchial cell line able to form epithelium. In this way it will be possible to clarify whether the effect of the correctors on the mutated protein observed in our experiments is due to the experimental conditions or whether the differentiation and polarization of the epithelium may play a role in modifying the properties of the CFTR rescued channels.

## Supplementary information

Supplementary information.
