# Supplementary material for: Correctors modify the bicarbonate permeability of F508del-CFTR
Source: Sci Rep. 2020 May 21;10:8440. doi: 10.1038/s41598-020-65287-4 (PMC7242338; doi:10.1038/s41598-020-65287-4)
Supplement: Supplementary file 1 — Supplementary information. [file 41598_2020_65287_MOESM1_ESM.docx]

**Correctors modify the bicarbonate permeability of F508del-CFTR**

**Supplementary data**

**Michele Fiore , Cristiana Picco and Oscar Moran**

*Istituto di Biofisica, Consiglio Nazionale delle Ricerche. Via De Marini, 6, 16149, Genova, Italy*

**The flux of bicarbonate from ammonium post-pulse alkalinization**

Experimental data were fitted with the function:

$pH\left( t \right)=pH\left( 0 \right)+\Delta pH\left( 1-exp\left( -tk \right) \right)$ (1)

where

$\Delta\left( pH \right)=pH\left( \right)-pH\left( 0 \right)$ (1a)

i.e. the difference between the asymptotic and the initial pH values, respectively.

According to the equation of Henderson-Hasselbach, for the bicarbonate/carbonic acid system we have:

$pH={pK}_{a}+log\left( \frac{Bic}{CA} \right)$ (2)

where *pK_a_* = 6.1, *Bic* is the concentration of bicarbonate, and the concentration of carbonic acid is *CA = k_CO2_ p_CO2_* ; *k_CO2_* = 0.03 (mmol/L)/mmHg is a constant including the solubility of carbon dioxide.

For the solution equilibrated with 5% CO_2_ (*f_CO2_* = 0.05), at 25°C we have a *p_H2O_* = 19.8 mmHg. Thus, at a barometric pressure of 760 mmHg, $p_{CO2}=f_{CO2}*\left( 760-19.8 \right)=37.05$ mmHg, and therefore, *CA* = 1.11 mM. Substituting the known values in (2), we obtain:

$pH=6.1+log\left( Bic \right)-log\left( 1.11 \right)=6.059+log\left( Bic \right)$ (3)

and substituting (3) in (1a):

$\Delta pH=6.059+log\left( {Bic}_{\infty} \right)-6.059-log\left( {Bic}_{0} \right)=log\left( \frac{{Bic}_{\infty}}{{Bic}_{0}} \right)$ (4)

and substituting (3) and (4) in (1):

$log\left( Bic\left( t \right) \right)=log\left( {Bic}_{0} \right)+\left[ log\left( \frac{{Bic}_{\infty}}{{Bic}_{0}} \right) \right]\left( 1-exp\left( -tk \right) \right)$ (5)

solving (5) for *HCO_3_^-^*:

$Bic\left( t \right)={Bic}_{\infty}\left( \frac{{Bic}_{\infty}}{{Bic}_{0}} \right)^{-exp\left( -kt \right)}$ (6)

The flux of bicarbonate is the derivative of (6):

$\frac{\text{d}Bic\left( t \right)}{\text{ d}t}={Bic}_{\infty}\left( \frac{{Bic}_{\infty}}{{Bic}_{0}} \right)^{-exp\left( -kt \right)}kexp\left( -kt \right)ln\left( \frac{{Bic}_{\infty}}{{Bic}_{0}} \right)$ (7)

and the initial flux, at *t* = 0 is:

$\frac{\text{d}Bic\left( t \right)}{\text{ d}t}\vee{}_{t=0}=k{Bic}_{0}ln\left( \frac{{Bic}_{\infty}}{{Bic}_{0}} \right)$ (8)

**Table S1.** Halide transport expressed as the initial quenching rate (QR), the HCO_3_^-^ flux (J_bic_) and the bicarbonate/iodide transport ratio, measured in cells transfected with p.F508del-CFTR, untreated, and incubated with VX809, VX661, Corr4a, and the combination of VX809 and Corr4a (Comb). Data is the average ± SEM (number of independent experiments).

|  | **F508del** | **+VX809** | **+VX661** | **+Corr4a** | **+Comb** | **WT** |
| --- | --- | --- | --- | --- | --- | --- |
| **QR (ms^-1^)** | 4.6 ± 0.8 | 47.0 ± 2.6 | 39.4 ± 2.3 | 29.2 ±1.4 | 95.5 ± 2.9 | 62.9 ± 1.4 |
|  | (12) | (12) | (9) | (12) | (12) | (10) |
| **J_bic_ (µM / s)** | 2.4 ± 1.2 | 25.8 ± 4.1 | 21.5 ± 1.7 | 17.0 ± 2.6 | 45.9 ± 10.0 | 14.5 ± 2.2 |
|  | (10) | (12) | (9) | (10) | (8) | (10) |
| **HCO_3_^-^/I^-^ transport ratio** | 0.52 ± 0.28 | 0.61 ± 0.09 | 0.55 ± 0.05 | 0.58 ± 0.09 | 0.52 ± 0.11 | 0.23 ± 0.04 |

**Table S2.** Comparison of the QR data presented in table S1. Data represents the P of a Student's t-test.

|  | **F508del** | **+VX809** | **+VX661** | **+Corr4a** | **+Comb** |
| --- | --- | --- | --- | --- | --- |
| **+VX809** | << 0.0001 |  |  |  |  |
| **+VX661** | << 0.0001 | 0.0398 |  |  |  |
| **+Corr4a** | << 0.0001 | << 0.0001 | 0.0020 |  |  |
| **+Comb** | << 0.0001 | << 0.0001 | << 0.0001 | << 0.0001 |  |
| **WT** | << 0.0001 | << 0.0001 | << 0.0001 | << 0.0001 | << 0.0001 |

**Table S3.** Comparison of the J_bic_ data presented in table S1. Data represents the P of a Student's t-test.

|  | **F508del** | **+VX809** | **+VX661** | **+Corr4a** | **+Comb** |
| --- | --- | --- | --- | --- | --- |
| **+VX809** | 0.0003 |  |  |  |  |
| **+VX661** | 0.0269 | 0.0001 |  |  |  |
| **+Corr4a** | 0.0227 | << 0.0001 | 0.3452 |  |  |
| **+Comb** | 0.4739 | 0.0002 | 0.0875 | 0.1735 |  |
| **WT** | 0.0162 | 0.0032 | 0.0448 | 0.0450 | 0.0234 |
